# Gastrin activates autophagy and increases migration and survival of gastric adenocarcinoma cells

**DOI:** 10.1186/s12885-017-3055-5

**Published:** 2017-01-21

**Authors:** Shalini V. Rao, Guri Solum, Barbara Niederdorfer, Kristin G. Nørsett, Geir Bjørkøy, Liv Thommesen

**Affiliations:** 1 Department of Cancer Research and Molecular Medicine, Norwegian University of Science and Technology (NTNU), Trondheim, Norway; 2 Department of Technology, NTNU, Trondheim, Norway; 3 CEMIR (Centre of Molecular Inflammation Research), NTNU, Trondheim, Norway; 4 The Central Norway Regional Health Authority, Stjørdal, Norway

**Keywords:** Gastrin, Gastric adenocarcinoma, Autophagy, STK11-PRKAA2-ULK1 signaling cascade, Cell migration, Cell survival, Chemoresistance

## Abstract

**Background:**

The peptide hormone gastrin exerts a growth-promoting effect in both normal and malignant gastrointestinal tissue. Gastrin mediates its effect via the cholecystokinin 2 receptor (CCKBR/CCK2R). Although a substantial part of the gastric adenocarcinomas express gastrin and CCKBR, the role of gastrin in tumor development is not completely understood. Autophagy has been implicated in mechanisms governing cytoprotection, tumor growth, and contributes to chemoresistance. This study explores the role of autophagy in response to gastrin in gastric adenocarcinoma cell lines.

**Methods:**

Immunoblotting, survival assays and the xCELLigence system were used to study gastrin induced autophagy. Chemical inhibitors of autophagy were utilized to assess the role of this process in the regulation of cellular responses induced by gastrin. Further, knockdown studies using siRNA and immunoblotting were performed to explore the signaling pathways that activate autophagy in response to gastrin treatment.

**Results:**

We demonstrate that gastrin increases the expression of the autophagy markers MAP1LC3B-II and SQSTM1 in gastric adenocarcinoma cells. Gastrin induces autophagy via activation of the STK11-PRKAA2-ULK1 and that this signaling pathway is involved in increased migration and cell survival. Furthermore, gastrin mediated increase in survival of cells treated with cisplatin is partially dependent on induced autophagy.

**Conclusion:**

This study reveals a novel role of gastrin in the regulation of autophagy. It also opens up new avenues in the treatment of gastric cancer by targeting CCKBR mediated signaling and/or autophagy in combination with conventional cytostatic drugs.

**Electronic supplementary material:**

The online version of this article (doi:10.1186/s12885-017-3055-5) contains supplementary material, which is available to authorized users.

## Background

Autophagy is an evolutionarily conserved process wherein the cytoplasmic components are degraded to provide cells with energy during starvation. Basal autophagy is necessary to maintain homeostasis and can be induced in response to cellular stress [1, 2]. The process of macroautophagy (herein referred to as autophagy) involves the engulfment of cytoplasmic material into *de novo* generated double membrane vesicle called autophagosomes. The isolated material is degraded after the fusion with ﻿the lysosomes [3]. The process of autophagy is orchestrated by a set of AuTophaGy-related genes (ATGs) that were first identified in yeast, but later shown to have orthologs in mammals [4]. Microtubule-associated protein 1 light chain 3 beta (MAP1LC3B-I/II/ LC3B) is lipidated when autophagy is induced and plays an essential role in the autophagosome formation [5]. Sequestosome 1 (SQSTM1/p62) facilitates the degradation of polyubiquitinated substrates by autophagy via the direct interaction with ubiquitinated proteins and MAP1LC3B located on the autophagosomal membrane [6]. MAP1LC3B and SQSTM1 are both produced and degraded in a coordinated manner during autophagy and therefore, are used as markers to study this process [7, 8].

The initiation of autophagy is orchestrated by the activity of the ULK1 (ATG1) kinase complex [9]. The activity of the ULK1 complex is positively regulated by the adenosine monophosphate-activated protein kinase (PRKAA2/AMPK) and inhibited by mammalian target to rapamycin (mTOR). This leads to balancing of cellular catabolic routes according to the innate needs of the cell. The activity of the ULK1 complex can be monitored by using specific antibodies,that recognize﻿﻿ the phosphorylation of ULK1 on Ser 555 or Ser 317 (stimulate the activity) or on ULK1 Ser 757 (inhibit the activity) [10–12].

The peptide hormone gastrin (G-17) is the central regulator in the maintenance and organization of the gastric mucosa and plays a pivotal role in gastric acid secretion in the stomach [13]. In addition, gastrin exerts growth-promoting effects in both normal and malignant gastrointestinal tissues in the oxyntic mucosa and the gastric epithelial cells [14]. Gastrin has been found to stimulate proliferation of cancer cell lines isolated from the stomach, pancreas and colon [15–17]. It has been reported to promote cellular responses such as migration, invasion and survival [18–20]. However, the role of gastrin in the progression of gastric adenocarcinoma is not completely understood. Nonetheless, hypergastrinemia in combination with *H. pylori* infections are considered to be a risk factor for the development of gastric adenocarcinomas [21].

We have previously reported that gastrin treatment of the pancreatic adenocarcinoma cell line AR42J resulted in differentially expressed genes which were annotated to cellular responses such as unfolded protein response (UPR)/ER stress and survival [22]. It is well established that UPR/ER stress is counteracted by increased autophagy [23]. Thus, we hypothesized that gastrin may be involved in the activation of autophagy in human gastric cancer cells. In this study, we find that gastrin treatment induces autophagy in the gastric adenocarcinoma cell lines AGS-Gr and MNK45, concomitant with the activation of the STK11-PRKAA2-ULK1 signaling cascade. Further, we demonstrate that gastrin treatment reduces the cytotoxic effect exerted by cisplatin. We propose that gastrin induced autophagy is in part responsible for the increased migration and chemoresistance of the AGS-Gr cells.

## Methods

### Cells

AGS (human gastric adenocarcinoma, ATCC, Rockville, MD) and AGS-Gr (stably transfected with CCKBR, gift from Prof. Andrea Varro, University of Liverpool) cells were grown in HAM’S F12 (GIBCO, 21765–029) supplemented with 10% FCS (GIBCO, 10270–106), 10 μg/ml penicillin-streptomycin and 2 μg/ml puromycin (GIBCO A11138-03). The MKN45 (human gastric adenocarcinoma) cell line was a gift from Prof. Susan A. Watson, University of Nottingham. The cells were grown in DMEM (GIBCO, 41965–039) with 4.5 g/l glucose, 10% FCS, 10 U/ml penicillin-streptomycin, and 1 μg/ml fungizone.

### Antibodies and siRNAs

The following antibodies were used for immunoblot analyses in the indicated final dilution: CCKBR (1:300), (Bioworld Technology, Cat no: BS3159); CCKBR (1:200) Abbiotech (Catno: 250659), MAP1LC3A-II (1:1000), (Cell signaling, Cat no:#3868); SQSTM1 (1:1000), (PROGEN Biotechnik GmbH,Cat no: GP62-C); ULK1 (1:500), (Cat no:#8054); p-ULK1Ser317 (1:500), (Cat no:#6887); p-ULK1Ser555 (1:500), (Cat no:#5869); p-ULK1 Ser757 (1:500), (Cat no:#6888) PRKAA2α (1:1000), (Cat no:#2532), p-PRKAA2α Thr172 (1:1000), (Cat no:#2535); STK11 (1:1000), (Cat no: #3482) p-RAPTOR Ser792 (1:800), (Cat no: #2083), RAPTOR (1:800), (Cat no: #2280) and p-STK11Ser 428 (1:1000), (Cat no:#3482), were all obtained from Cell Signaling; ACTA1 (1:5000), (Abcam, Cat no:8227); GAPDH (1:5000), (Abcam, Cat no:9484), PCNA (1:2000),(Abcam, Cat no:Ab29) and HRP-conjugated (1:5000) rabbit & mouse polyclonal antibodies, (DAKO E0453 and ISO76), Secondary Antibody (LICOR) Donkey anti-guinea pig (1:5000), (Cat no: P/N 926-32411), Goat anti-mouse (1:15000), (Cat no: P/N 925–32213); Goat anti-rabbit (1:5000), (Cat no: P/N 925–68070). The following siRNAs were used: siRNAs targeting CCKBR were obtained from (Invitrogen, Primer no: 250273C09, 250273C10 & 250273C011), siRNA STK11 (Thermo Scientific Cat no: S02349811) and ON-TARGET plus Non-Targeting Pool were obtained from (Dharmacon Cat no: D-001810-01-20). Gastrin (Sigma Cat no: SCP01050, G-17), Compound C (Millipore Cat no: 171260), Bafilomycin A1 (Sigma Cat no: B1 793), the CCKBR antagonist YM022 (Sigma Cat no: SML0220) and hydroxychloroquine (Sigma Cat no: HO915).

### Immunoblot analyses

Cells were cultivated without serum only during gastrin stimulation and harvested in 8 M Urea lysis buffer, 0.50% Triton-X 100, 0.1 M DTT, Protease inhibitor 1 & 2 Sigma Cat no: P8340) and Phosphatase inhibitors (Roche). Phosphorylated proteins were harvested in 1 M Tris–HCl, pH 8.0, 1 M KCl, 0.5 M EDTA, 87% Glycerol, 100% NP-40. A saturation curve for the proteins was estimated by loading 10–80 μg of protein for immunoblotting. We loaded 35 μg of total cell lysate protein to avoid saturation and assessed ACTA1, MAP1LC3B and SQSTM1 levels in the linear protein detection range. The immunoblotting procedure was performed as previously described [24]. Secondary antibodies were visualized by using the Super Signal West Femto Maximum Sensitivity Substrate (Pierce, ThermoScientific, Cat no: #34096). Both fluorescence and chemiluminescence was visualized using ODYSSEY^®^ Fc Imaging System. Image Studio software was used to quantify and adjust contrast on the immunoblots.

### Confocal microscopy

Cells (10 000 cells in 200 μl medium with 10% FBS) were seeded on Lab-Tek™ chambered coverglass with 8 wells (NUNC, Thermo Scientific) and left overnight. Cells were serum starved and treated with gastrin (10 nM) and Baf A1 (100 nM) for 4 h. Cells were fixed (4% paraformaldehyde in PBS) for 10 min, washed (PBS x 2) and permeabilized (ice-cold MeOH) for 10 min on ice and washed (PBS x 2). Cells were stained with Draq-5 (1:1000), (Biostatus, DR05500) for 7 min, washed and stored at 4 °C over night before confocal microscopy. The cells were immunostained after a 1 h blocking using 3% goat serum in PBS followed by incubation of the properly diluted primary antibodies in 1% goat serum in PBS. Unbound antibodies were removed by washing 5 times 5 min incubations in PBS before fluorescent dye labelled secondary antibodies were applied according to the species origin of the primary antibody. Confocal microscopy studies were performed with a Zeiss Axiovert 100-M inverted microscope equipped with an LSM 510 laser-scanning unit and a 1.4 numerical aperture × 63 Plan-Apochromat oil immersion objective. Laser power was typically 30% and the pinhole was set to 0.8–1.2 μm. Multitracking was used for dual color imaging at 488 nm and 647 nm.

### Transfection

300 000 cells were seeded into 6-well plates and cultured for 24 h before transfection with siRNA using Metafectene Pro (Biontex Cat no: T040-1.0). The media was replaced 6 h after transfection. 81 nM siRNA and 12 ul Metafectene was used per well. The cells were left undisturbed for 48 h post transfection. AGS-Gr and MKN45 cells were transfected twice with siRNA targeting CCKBR on following days to obtain a better knockdown.

### Cell viability and proliferation assays

To measure changes in cell viability, gastrin and BafA1 treated - cells were stained using the Apotest FITC kit (Nexins Research Cat no: N1470036). The cells were incubated with annexin V FITC (0.2 l g/mL in 19 annexin binding buffer) for 1 h on ice. Propidium iodide (PI) (1.4 g/mL) was added 5 min prior to data acquisition using an LSRII flow cytometer (BD Biosciences). Cells negative for both annexin V and PI staining were considered viable. The number of metabolic active, viable cells were quantified using XTT assay by using TACS XTT cell proliferation assay kit (Trevigen Cat no: 481-025-k) according to manufactures instructions. The cells were stimulated with gastrin (10 nM) for 2 h, before cisplatin was added. Hydroxy-chloroquine (HCQ) was used at a final concentration of 10 μM. The viable cells were assessed at 48 h after cisplatin treatment. Autophagy was blocked for 12 h. The ULK1 inhibitor SBI-0206965 was used with 5 μM final concentration and added to cells alone or together with gastrin for 24 h and 48 h before absorbance was determined using a microplate Reader (BIORAD) at dual wavelength; 490 nm and 620 nm.

### Migration assay

The xCELLigence® DP system (Roche Diagnostics GmbH, Germany) was used to study migration as previously described [25]. Briefly, AGS-Gr cells were seeded into (5.0 x 10^4^ cells/well) the CIM-Plate 16 (Roche). The lower chamber contained 1 nM gastrin alone or in combination with ULK1 inhibitor SBI-0206965 (10 μM) final concentration) (Apex Biosciences A8715), Compound C (Millipore), HCQ (20 μM), BafA1 (100nM). Cell migration was monitored every 15 min on a RTCA DP instrument for 24 h. Data analysis was carried out using RTCA Software 1.2 supplied with the instrument.

### Caspase assay

Caspase activity was measured by using the Caspase-Glo 3/7 assayfrom Promega (Madison, WI) according to the manufacturer’s descriptions. Luminescence was measured using Wallac 1420 Victor3 plate reader (Perkin Elmer). The AGS-Gr cells were seeded out into white-walled 96-well plates (Perkin Elmer) and the cells were treated with Gastrin (10 nM), cisplatin (10 μM) for 72 h. BafA1 (100 nM) was added 16 h prior to termination of the assay.

### Statistics

Statistical values were expressed as mean ± standard deviation (SD). Statistical analysis was performed by the two-tailed Student *T*-test. P values < 0.05 was considered statistically significant and is labelled with P- values: *** ≤ 0.01 ** ≤ 0.02, * ≤ 0.05.

## Results

### Gastrin induces autophagy in gastric adenocarcinoma cells

**Fig. 1:**
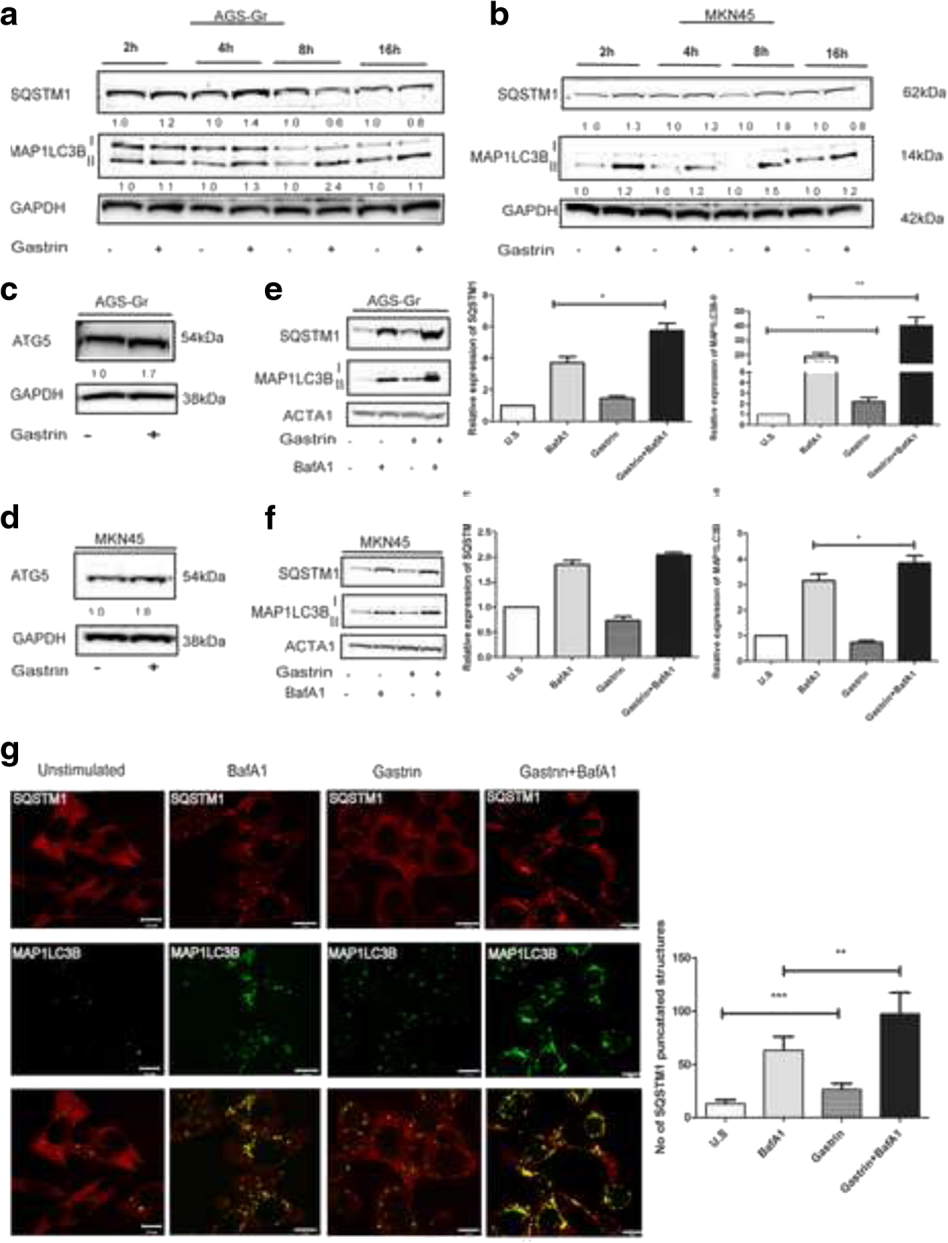
Gastrin upregulates autophagy markers in gastric adenocarcinoma cells. **a** and **b** AGS-Gr and MKN45 cells treated with gastrin (10 nM) for 2–16 h. The expression of MAP1LC3B-I/II and SQSTM1 is shown by immunoblotting. The images represent one of two independent experiments (**c** and **d**) Cells treated with gastrin for 4 h. The expression of ATG5 is shown by immunoblotting. **e** and **f** AGS-Gr and MKN45 cells treated with BafA1 (100 nM) and gastrin for 8 h and 4 h, respectively. The expression of MAP1LC3B-II and SQSTM1 is shown by immunoblotting. The images represent one of three independent experiments. Graphs represents three independent experiments, mean +/− SEM (P- values: ** ≤ 0.02 and * ≤ 0.05) (**g**) AGS-Gr cells treated with BafA1 and gastrin for 4 h. Cells were stained for MAP1LC3B (Alexa 488) and SQSTM1 (Alexa 647). The images were processed using IMAGE J software. 300 cells were manually counted for SQSTM1 puncatated structures in the cytosol. U.S. = untreated cells. The images represent one of three independent experiments. Graphs represent three independent experiments; mean +/− SEM (P- value: *** ≤ 0.02)

Our transcriptome analysis of differentially expressed genes in the pancreatic adenocarcinoma AR42J cells revealed that gastrin upregulates the mRNA level of autophagy related genes (e.g. *Sqstm1 and beclin 1* (E-MTAB-1268 and GSE32869)) [22], suggesting that gastrin may induce autophagy. Several recent studies have identified elevated autophagy in gastric cancer [26, 27]. Thus, we tested if gastrin could induce autophagy and if this could contribute to tumor progression. We utilized two gastric cancer cell lines, MKN45 that expresses the CCKBR endogenously and the AGS-Gr that stably overexpresses the CCKBR (Additional file 1: Figure S1) [28, 29]. The two cell lines were treated with 10 nM gastrin and the protein levels of MAP1LC3B-II and SQSTM1 were assessed by immunoblotting. In both the cell lines, gastrin enhanced the protein level of MAP1LC3B-II and SQSTM1 in a time dependent manner (Fig. 1a & b). The protein level of autophagy related protein ATG5 was also slightly elevated after gastrin treatment in AGS-Gr and MNK45 cells at 4 h and 2 h, respectively (Fig. 1c & d). Since MAP1LC3B-II and SQSTM1 are constantly degraded by autophagy, an increased level of the proteins could result both from elevated synthesis and/or reduced autophagic degradation. To discriminate between these two possibilities, gastrin treatment was performed in the presence of the lysososmal inhibitor Bafilomycin A1 (BafA1). As expected, treatment of cells with BafA1 alone caused an accumulation of the MAP1LC3B and SQSTM1 proteins (Fig. 1e & f). Interestingly, in the AGS-Gr cells treated with gastrin + BafA1, we found a significant increase in MAP1LC3B-II and SQSTM1 levels compared to the level in cells treated with BafA1 alone (Fig. 1e; Additional file 1: Figure S2 (a) & (b)). In the MKN45 cells, gastrin + BafA1 treatment caused a small but consistent enhancement in the level of MAP1LC3B-II, while the effect on SQSTM1 was not statistically significant (Fig. 1f). The stronger gastrin response demonstrated in the AGS-Gr cells might be attributed to the higher expression of the CCKBR in these cells (Additional file 1: Figure S1).

To substantiate the above findings, we examined the cellular localization of SQSTM1 and MAP1LC3B-II using immunostaining. In AGS-Gr cells, we found a significant increase in the number of cytoplasmic SQSTM1-stained punctuated structures 4 h after gastrin treatment (Fig.1g & Additional file 1: Figure S3). In line with the immunoblotting analyses, the number of SQSTM1 structures were higher when combined with the lysosomal inhibitor and gastrin compared to each treatment alone.

**Fig. 2:**
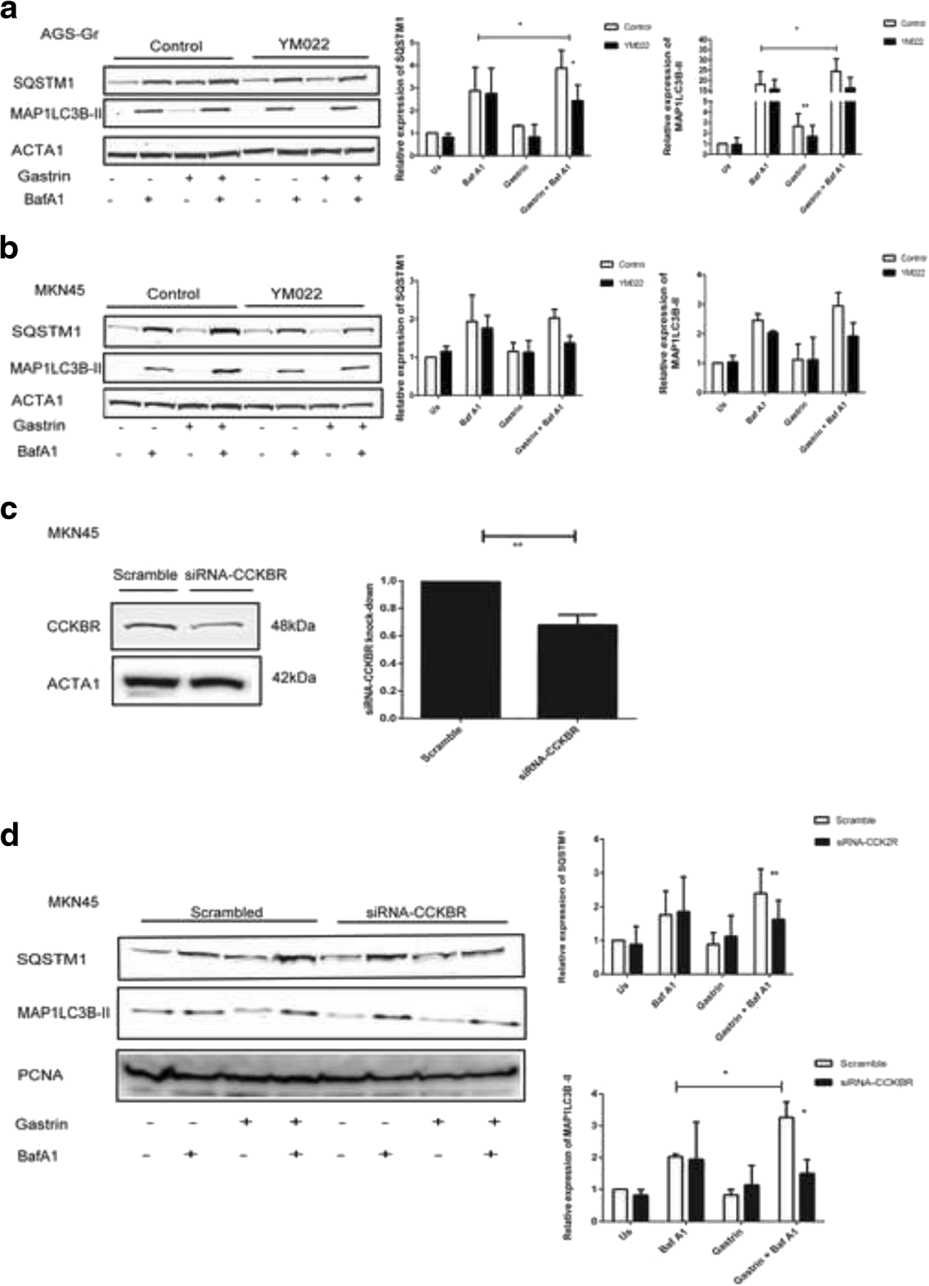
Gastrin induced autophagy is mediated via the CCKBR: (**a** and **b**) AGS-Gr and MKN45 pretreated overnight with YM022 (100 nM) before treatment with BafA1 and gastrin for 4 h. The expression of MAP1LC3B-II and SQSTM1 is shown by immunoblots representing one of three independent experiments. (**c**) MKN45 cells transfected with siRNA CCKBR. Protein expression of CCKBR was analyzed by immunoblotting. (**d**) MKN45 cells transfected with siRNA CCKBR and treated with BafA1 and gastrin. MAP1LC3B-II and SQSTM1 expression is shown by immunoblotting. The image represents one of three independent experiments. Bar graphs (**a**, **b**, **c** and **d**) show mean +/− SEM, (*n* = 3, *P*- value ** ≤ 0.02 and * ≤ 0.05)

The activity of CCKBR can be targeted by using the chemical inhibitor YM022 [30]. In line with a role of gastrin in inducing autophagy, pretreatment with YM022 resulted in a decrease in the gastrin induced MAP1LC3B-II level by approximately 50% and 35% in AGS-Gr and MKN45 cells, respectively (Fig. 2a & b; compare gastrin + BafA1 treated cells +/− YM022). Consistently, the presence of YM022 reduced gastrin induction of SQSTM1 protein by 45% and 30% in the AGS-Gr and MKN45 cells, respectively. Likewise, the knockdown of CCKBR in MKN45 cells using siRNA (Fig. 2c) significantly reduced gastrin induced autophagy (i.e. downregulation of SQSTM1 and MAP1LC3B-II levels) (Fig. 2d). Collectively, the data demonstrates that the gastric adenocarcinoma cell lines display an increased autophagy in response to gastrin in a CCKBR dependent manner.

Blocking of autophagy reduces gastrin-induced migration

**Fig. 3:**
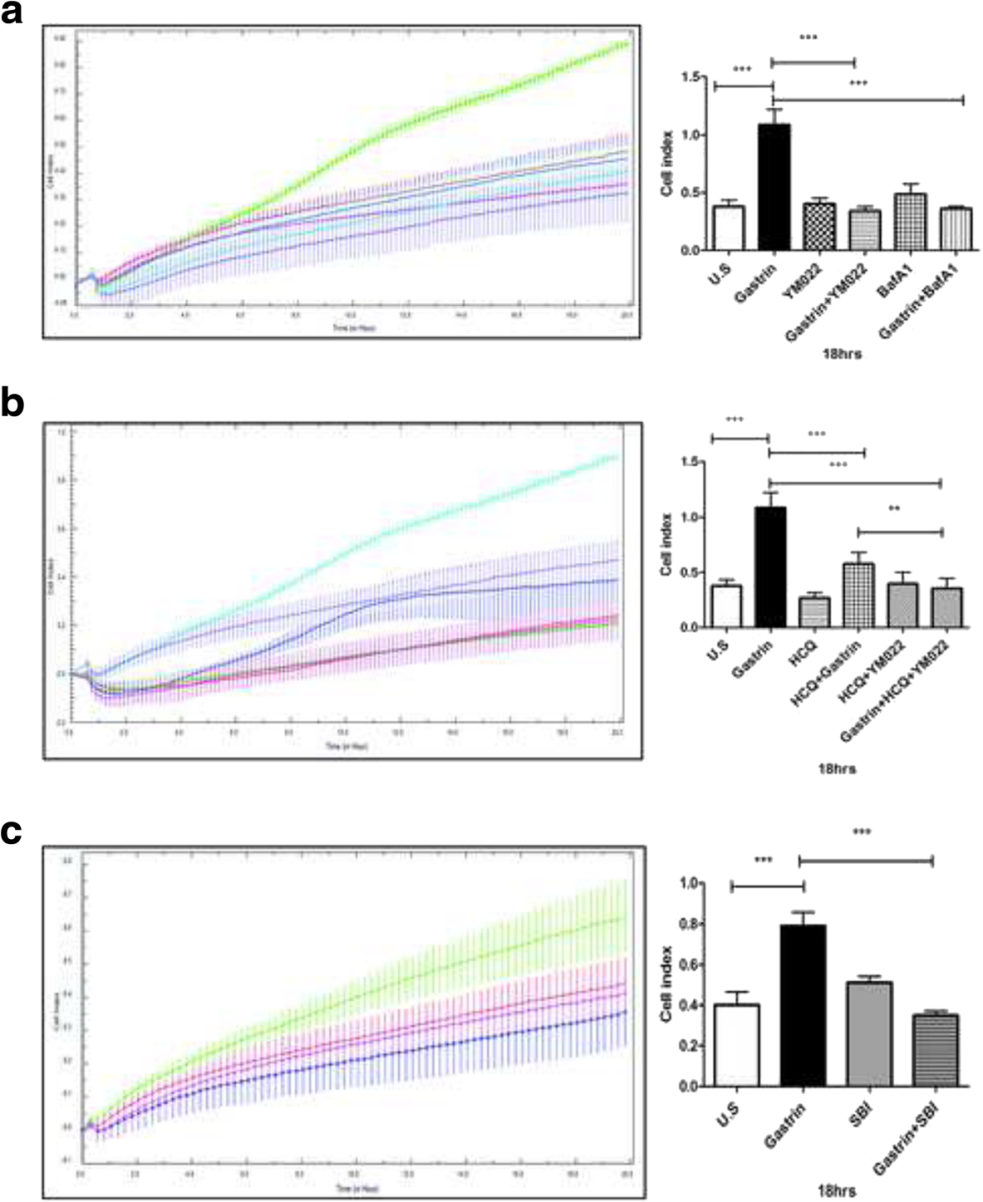
Gastrin induced migration is dependent on autophagy. ﻿(**a**) AGS-Gr cells treated with gastrin (1 nM), BafA1 (100 nM) and YM022 (50 nM). Migration was monitored real-time for 24 h using xCELLigence technology. Untreated (blue), gastrin (green), YM022 (pink), gastrin + YM022 (violet), BafA1 (red), gastrin + BafA1 (light blue). Graphs represent of one of three independent experiments. (**b**) AGS-Gr cells treated with gastrin, YM022 and HCQ (20 μM)). Untreated (violet), Gastrin (light blue), HCQ (pink), HCQ + gastrin (dark blue), HCQ + YM022 (green), HCQ + YM022 + gastrin (red). (**c**) Gastrin induced migration is dependent on ULK1. AGS-Gr cells were treated with gastrin and ULK1 inhibitor SBI-026965 (SBI) (10 μM) Untreated (red), gastrin (green), SBI-026965 (blue), gastrin + SBI-026965 (pink). (**a**, **b** and **c**) Graphs represent of one of three independent experiments. Bar graphs represent mean +/− SEM (*n* = 3, *P*-value*: ≤ 0.05, ** ≤ 0.01, *** ≤ 0.001)

Others and we have previously reported increased migration and cell survival in response to gastrin [18, 24, 31]. In the present study, we assessed the influence of gastrin induced autophagy on both cell migration and cell survival. Autophagy has an established role in cell survival [32]. Recently it was also identified as an important factor in the regulation of migration of Ras transformed MCF10A cells [33]. Thus, we tested if autophagy contributes to the gastrin induced migration. Inhibiting lysosomal degradation in the AGS-Gr cells with BafA1 or hydrochloroquinone (HCQ) significantly reduced gastrin-induced migration by approx. 60% (BafA1) and 40% (HCQ) at 18 h (Fig. 3a & b). When the CCKBR antagonist YM022 was added to the cells, we found that gastrin induced migration was inhibited (Fig. 3a). The antagonist by itself did not influence migration. Further, YM022 was utilized in combination with HCQ and gastrin, we show that the migration was further reduced significantly (30%) (Fig. 3b; compare HCQ + gastrin versus HCQ + gastrin + YM022).

The ULK1 inhibitor SBI-026965 was used to evaluate the role of autophagy in induced migration. We find that inhibition of ULK1 (ATG1) resulted in a reduction of gastrin induced migration by 50% (18 h) compared to untreated cells (Fig. 3c). Taken together, our results suggest that autophagy plays a crucial role in gastrin induced migration in the AGS-Gr cells.

## Blocking of autophagy reduces gastrin-induced cell survival

**Fig. 4:**
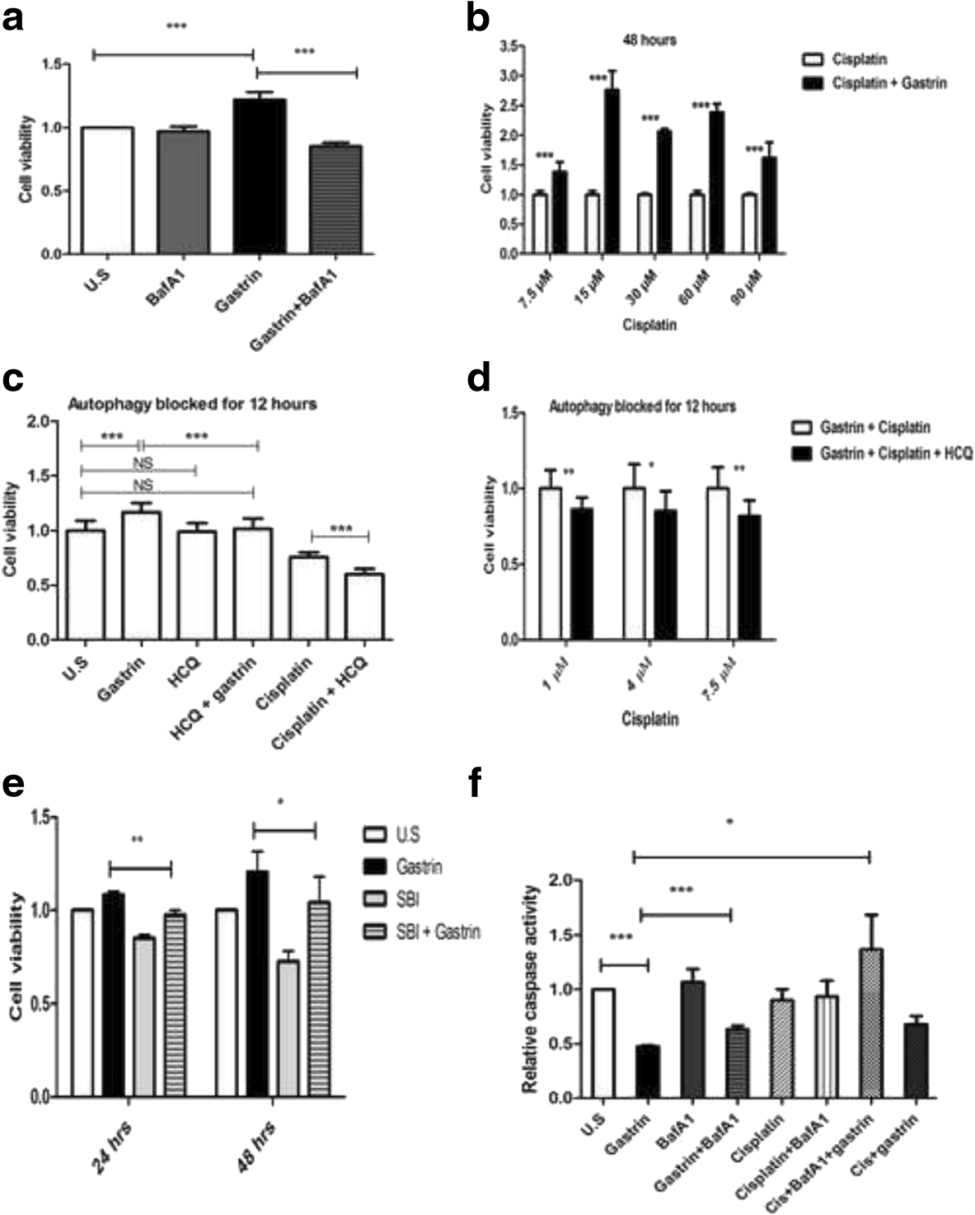
Gastrin induced survival is dependent on autophagy. (**a**) AGS-Gr cells treated with gastrin +/− BafA1 for 18 h. Cell viability was assessed using annexin V-PI staining and flow cytometric analyses. The viability of untreated cells (U.S.) is set to 1.0. (**b**) Gastrin reduces cisplatin induced cell death. AGS-Gr cells treated with increasing doses of cisplatin (7.5-90 μM) in presence or absence of gastrin (10 nM). Cell viability assessed by XTT assay; the viability of cisplatin treated cells is set to 1.0 for each concentration (**c**) AGS-Gr cells treated with gastrin (2 h) with subsequently treatment with cisplatin (7.5 μM) for 36 h. Autophagy was blocked for 12 h using HCQ. Cell viability was determined by XTT assay; the viability of untreated cells (U.S.) set to 1.0. (**d**) Cells treated with gastrin for (2 h) and subsequently with increasing concentrations of cisplatin (1–7.5 μM) for 36 h before autophagy was blocked for 12 h. (**e**) Gastrin induced survival is dependent on ULK1. AGS-Gr cells treated with ULK1 inhibitor SBI-026965 (SBI) (5 μM) for 24 h and 48 h in the presence or absence of gastrin (10 nM). (**f**) Caspase activity performed with AGS-Gr cells pretreated with gastrin (2 h), followed by cisplatin (10 μM) treatment for 72 h. Autophagy was blocked for 12 h with BafA1. Bar graphs (**a**, **b** **c**, **d**, **e** and **f**) represent SEM (*n* = 3, *P*-value: * ≤ 0.05, ** ≤ 0.01, *** ≤ 0.001)

Induced autophagy represents a survival mechanism in tumour cells that may enable them to survive during stressful conditions including exposure to cytostatic drugs [27]. We examined whether gastrin induced autophagy was essential for the increased cell survival. Consistent with previous reports, gastrin caused a slight, but consistent and significant increase in the number of viable cells under serum-free conditions using annexin-PI staining (Fig. 4a). The lysosomal inhibitor BafA1 did not affect the viability of AGS-Gr cells by itself, but interestingly, adding BafA1 together with gastrin reduced the pro-survival effect of gastrin by 30% (Fig. 4a & Additional file 1: Figure S4a). The data suggests that induced autophagy contributes to enhanced cell survival in response to gastrin.

Cisplatin is used in the treatment of gastric adenocarcinomas [34, 35], and we tested whether gastrin induced autophagy could modify the cellular response to cisplatin in vitro. Initially, AGS-Gr cells were treated with increasing concentrations of cisplatin (1–90 μM) in the presence and absence of gastrin for 24 h, 48 h and 72 h. The numbers of surviving cells were determined by their metabolic activity (XTT assay). We found that gastrin treatment reduced the sensitivity towards cisplatin (Fig. 4b & Additional file 1: Figure S4 (c) & (d)). To explore if this gastrin induced survival was due to the induced autophagy, we performed the experiments in the presence of the autophagy inhibitor HCQ. Interestingly, addition of HCQ reduced the survival effect of gastrin (Fig. 4c) and the viability of cells treated with gastrin + cisplatin (Fig. 4d). HCQ diminished the survival effect induced by gastrin with increasing concentrations (1 μM, 4 μM and 7.5 μM) of cisplatin (Fig. 4d). This indicates that the survival effect of gastrin involves the induction of autophagy. Notably, HCQ did not affect cell viability by itself (Fig. 4c). As anticipated, HCQ treated cells showed an accumulation of both SQSTM1 and MAP1LC3B-II (Additional file 1: Figure S4 (e)). Next, we treated AGS-Gr cells with the ULK1 inhibitor SBI in the presence of BafA1 + gastrin. This resulted in reduced accumulation of SQSTM1 and MAP1LC3B-II (Additional file 1: Fig. S4 (f)). Further, AGS-Gr cells were treated with gastrin +/− SBI and cell viability determined by XTT assay. Consistently, gastrin alone increased the cell viability, but in the presence of SBI, cell viability was reduced by approx. 15% compared to cells treated with gastrin alone, at both 24 and 48 h (Fig. 4e). As previously reported the inhibitor on its own reduced cell viability [36].

To establish the link between gastrin induced autophagy and apoptosis, we examined the activation of caspase 3/7 in the AGS-Gr cells. Initially, the cells were treated with gastrin +/− BafA1. Gastrin treatment alone reduced the induction of caspase 3/7 activity (Fig. 4f), this coincides with a study in the gastrin responsive AR42J cells [37]. The reduced activation of caspases in the presence of gastrin was counter acted by BafA1 treatment (Fig. 4f). When, the cells were treated with gastrin in the presence or absence of cisplatin (autophagy blocked 16 h), we find that the inhibition of autophagy increased the activation of caspase 3/7 (Cisplatin + gastrin + BafA1 versus Cisplatin + gastrin) (Fig.4f). However, activation of caspases in the presence of cisplatin alone or in combination with BafA1 was found not to be significant. Further, when gastrin was added to cells treated with cisplatin, we observed a reduced caspase activity, suggesting that gastrin exerts a cytoprotective effect on these cells (Fig. 4f). Collectively, these results suggest that gastrin induced autophagy is linked to the anti-apoptotic effect exerted by gastrin.

## Gastrin activates the STK11–PRKAA2-ULK1 signaling pathway

**Fig. 5:**
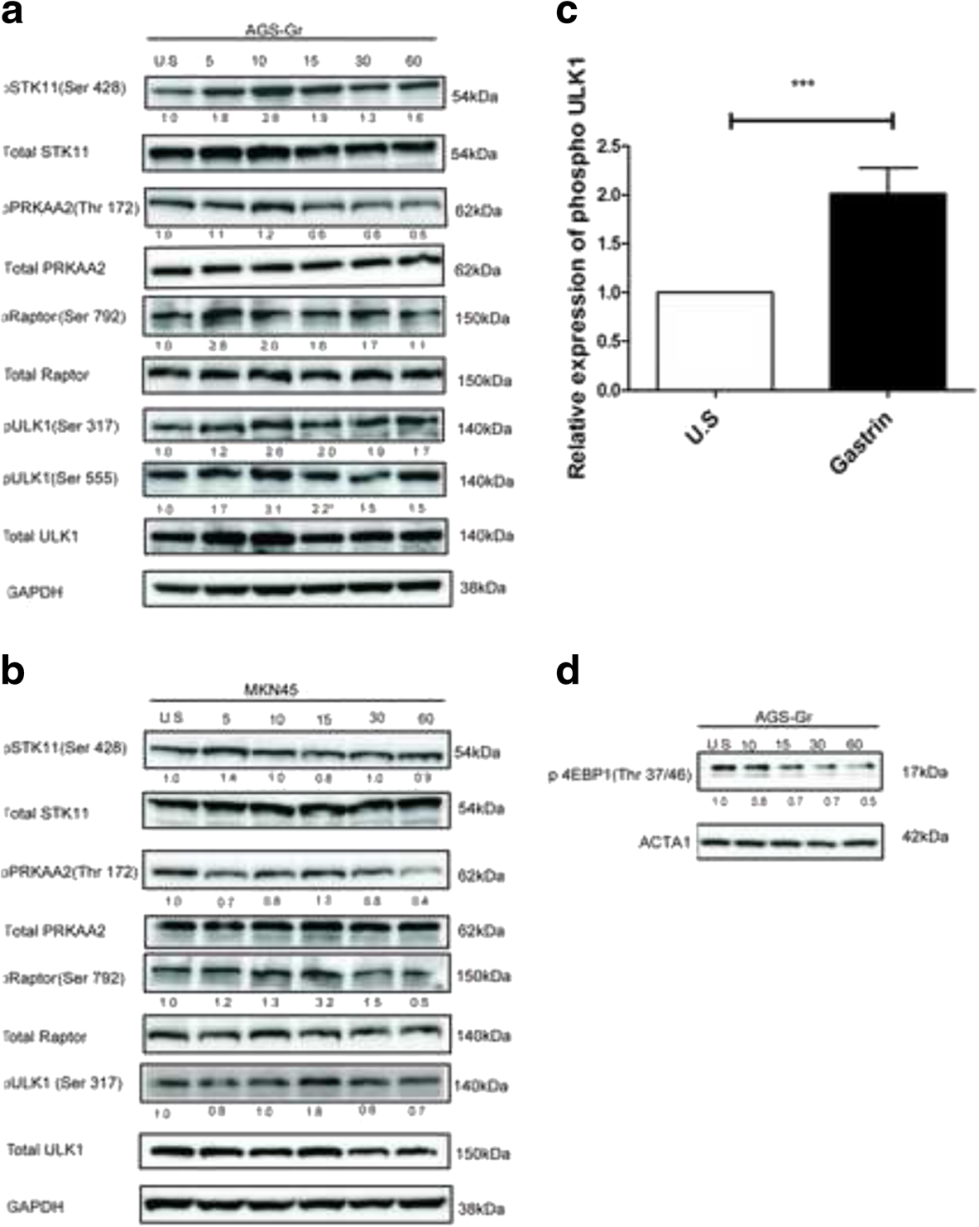
Gastrin induces phosphorylation of the LKB-1-PRKAA2-ULK1 pathway. (**a**, **b** and **c**) Cells were serum starved overnight and treated with gastrin (10 nM). Phosphorylated STK11 (Ser 428), PRKAA2 (Thr 172), ULK1 (Ser 317 & Ser 555), Raptor (Ser 792) and 4EBP1 (Thr 37/46) are shown by immunoblotting. Data was normalised to total protein and immunoblots shown represent one of three independent experiments. Bar graphs (**c**) represent SEM (*n* = 3, *P*-value: *** ≤ 0.01)

**Fig. 6:**
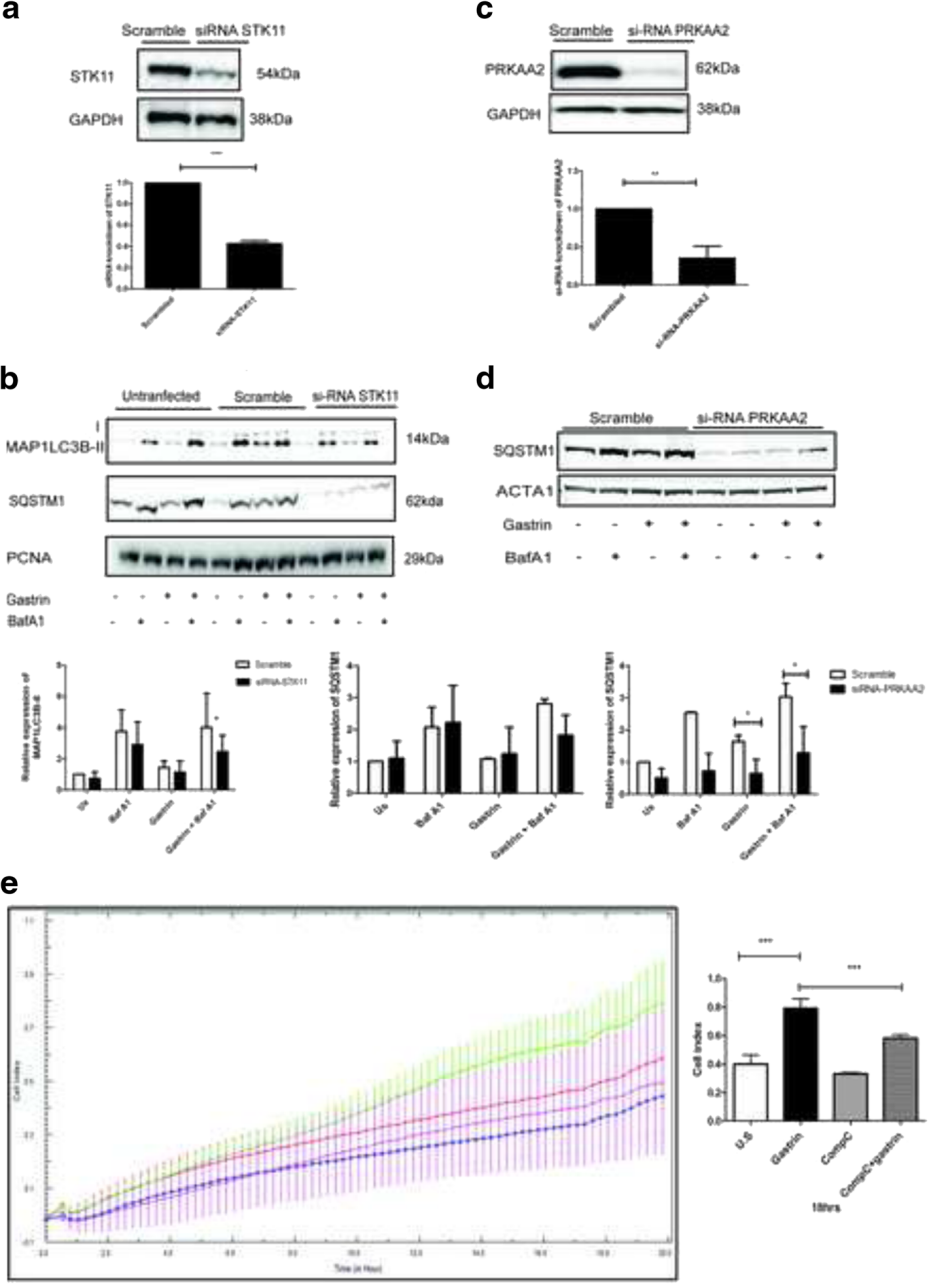
Knockdown of STK11 and PRKAA2 downregulates gastrin mediated autophagy. (**a**) Knockdown of STK11 in AGS-Gr cells. (**b**) AGS-Gr cells transfected with siRNA STK11 for 48 h before BafA1 and gastrin treatment (4 h). Expression of SQSTM1 and MAP1LC3B-II are shown by immunoblotting representing one of four independent experiments. Bar graphs (**a** and **b**) show mean +/− SEM (*n* = 3, *P*- value ** ≤ 0.02 and * ≤ 0.05). (**c**) Knockdown of PRKAA2 in AGS-Gr cells. (**d**) AGS-Gr cells transfected with siRNA PRKAA2 and treated with BafA1 and gastrin (4 h). Expression of PRKAA2 and SQSTM1 is shown by immunoblotting representing one of three independent experiments. (**e**) Gastrin induced migration is dependent on PRKAA2. AGS-Gr cells treated with gastrin (1 nM) and PRKAA2 inhibitor Compound C (10 μM) for 24 h. Migration was monitored using xCELLigence technology. Untreated (red), Comp C (blue), gastrin + Comp C (pink), gastrin (green). Bar graphs (**a**, **b**, **c**, **d** and **e**) show mean +/− SEM (*n* = 3, *P*-value*: ≤ 0.05, ** ≤ 0.01, *** ≤ 0.001)

The data presented above are consistent with a gastrin induced autophagy that stimulates migration and potentiates cell survival. The ULK1 kinase is the master regulator of autophagy by coordinating the initial steps of autophagosome formation. Since the data suggests that gastrin induces autophagy, we asked if this involves the activation of ULK1. We have recently demonstrated that gastrin induces STK11 Ser 428 phosphorylation in AGS-Gr and MKN45 cells [25], indicating that the STK11-PRKAA2-ULK1 signalling pathway might be involved in gastrin mediated induction of autophagy. Thus, we treated cells with gastrin and examined the phosphorylation of PRKAA2 by immunoblotting. In line with a gastrin induced activation of STK11, the phosphorylation of the STK11 targeted site in PRKAA2 (Thr 172) was transiently elevated in both AGS-Gr and MKN45 cells (Fig. 5a & b). Concurrent with an elevated activity of PRKAA2, we found that gastrin treatment also increased the phosphorylation of the autophagy activating sites of the ULK1 complex (Ser 317 and Ser 555) (Fig 5a & c). These data indicate a direct signalling pathway mediated by gastrin/CCKBR to the activation of ULK1 via increased activity of STK11 and PRKAA2. However, ULK1 may in addition be regulated indirectly by the same pathway, ie. if the gastrin induced PRKAA2 activity results in reduced mTOR activity. To further unravel signalling pathways involved in autophagy, we examined gastrin mediated phosphorylation of Regulatory-associated protein of mTOR (Raptor) Ser 792, which is known to inhibit mTOR activity. As shown in (Fig 5a & b), gastrin induced the phosphorylation of Raptor Ser 792. In the AGS-Gr cells, the phosphorylation of Raptor Ser 792 appeared as early as 5 min, and in the MKN45 cells at 15 min. Consistent with the activation of ULK1 on the autophagy activating sites (Ser 555 and Ser 317) we also found that the mTOR substrate 4EBP1 was less phosphorylated after gastrin treatment of the AGS-Gr cells (Fig. 5d). Collectively, these results suggest that the elevated autophagy in response to gastrin treatment is both due to a direct effect on PRKAA2, which induces the ULK1 activity, and the indirect effect via reduced mTOR activity. Additionally, AGS-Gr cells were transfected with siRNA towards STK11 and subsequently treated with gastrin before the assessment of the autophagy markers. The protein level of STK11 was reduced by ~ 60% compared to cells transfected with non-targeting siRNA (Fig. 6a). As shown in Fig. 6b, we observed a reduction in gastrin induced expression of MAP1LC3B-II and SQSTM1 when STK11 was knocked down. Similarly, targeting PRKAA2 with siRNA or a chemical inhibitor (Comp C) resulted in the downregulation of SQSTM1 (Fig. 6c, d & Additional file 1: Figure S5). Taken together, our results are congruent with a gastrin induced autophagy involving the activation of STK11–PRKAA2-ULK1 signaling pathway.

To elucidate a functional relationship between the gastrin-induced signalling cascades detailed above and migration, we treated AGS-Gr cells with the PRKAA2 inhibitor Compound C (Comp C). We found that Comp C decreased the gastrin-induced migration by approx. 40% (18 h) (Fig. 6e), indicating the involvement of the autophagy regulated signalling pathway. Together, these results suggest that the STK11 - PRKAA2 pathway controls autophagy and that this is important for the enhanced cell survival and migration in response to gastrin.

## Discussion

Gastrin exerts a growth promoting effect on several gastrointestinal cancer cells and a variety of neoplasms that express CCKBR, including neuroendocrine, pancreatic, medulla thyroid and lung cancer [38–40]. Interestingly, Hur et al. demonstrated that gastrin and CCKBR are expressed in approx. 50% of gastric carcinoma tissues, and patients with diffuse type of gastric carcinoma expressing both gastrin and CCKBR had poorer prognosis compared to those who were negative for both [41]. However, the role of gastrin in adenocarcinoma is still not completely understood, and whether gastrin acts as an autocrine/paracrine growth factor in gastric carcinoma is unclear. In the current study, we report that gastrin induces autophagy and increases cell migration and survival in vitro, and suggest that these molecular mechanisms may contribute to tumor progression of gastric cancer cells.

Several recent studies have indicated a role of autophagy and autophagy related proteins in the progression of gastric cancer [42, 43]. Immunohistochemistry analysis has identified an elevated staining intensity for autophagy related proteins such as Beclin1, Atg5, Atg8/ MAP1LC3B, and SQSTM1/p62 in gastric cancer tissue [26, 42]. Upregulation of MAP1LC3A was shown to correlate with an increase in Ki67 positive cells in gastrointestinal tumors [44]. Ge et al. demonstrated that *ATG5* was highly expressed in gastric cancer patients compared to healthy individuals, which was speculated to contribute to increased chemoresistance [45]. Autophagy is an incessant process, where numerous proteins including MAP1LC3B and SQSTM1 are constantly degraded. Thus, elevated levels of the proteins in a biopsy could result from both activation and inactivation of the process. Additionally, how autophagy could possibly be activated and contribute to gastric cancer development is currently not fully understood. We find that the peptide hormone gastrin induces autophagy in two gastric adenocarcinoma cell lines via the STK11-PRKAA2-ULK1 pathway. This signaling pathway may contribute to cancer development by both increasing cell survival and cell migration.

Loss of STK11 has been shown to correlate with increased cancer development. Nguyen and Liu et al. [46, 47] demonstrated that STK11 deficient melanoma cells have increased invasive properties compared to cells with normal amounts of STK11 protein. Contrarily, STK11 has been reported to be necessary for the survival of colorectal cancer cells, hepatocyte proliferation, liver regeneration and cell survival of liver tumors with constitutive activation of AKT [48, 49]. Our result establishes a functional role of STK11 in gastrin induced autophagy; the inhibition of downstream kinases PRKAA2 and ULK1 reduced gastrin-induced migration and survival. The role of autophagy in the migration of cancer cells is currently being investigated. Kenefic et al. found that autophagy is involved in the turnover of focal adhesion (FA), promoting FA disassembly and that this is dependent upon the autophagic receptor NBR1 [33]. Further, activation of autophagy in glioblastoma cells impairs the migration and invasion capacities via the downregulation of epithelial mesenchymal transition proteins such as SNAIL and SLUG [50]. Autophagy deficiency was found to increase SQSTM1/p62 levels, resulting in reduced E-cadherin expression, stabilization of the oncogenic protein Twist1 and promotion of cell migration, invasion and proliferation of human squamous cell carcinomas [51].

Gastrin is known to induce cell survival via several mechanisms [18, 52]. We find that gastrin partially counteracts the apoptotic effect of cisplatin, and blocking autophagy reduces the survival effect induced by gastrin. Xu et al. reported targeting autophagy sensitizes gastric cancer cells to oxaliplatin induced apoptosis [32]. Inhibiting lysosomal degradation by using BafA1 in combination with 5-Flurouracil (5-FU) resulted in decreased viability and colony forming capacity of SGC-7901 cells [27]. Kim et al. showed that cisplatin treatment induced phosphorylation of AMPK in the AGS cells, and that pharmacological inhibition/siRNA towards AMPK sensitized the cells to cisplatin induced apoptosis [53]. These results suggest that AMPK and autophagy contribute to a general chemo resistance and act in a cytoprotective manner in response to anti-cancer therapy.

Recently Egan et al., found that inhibition of ULK1 reduced the survival of A549 cells significantly, when combined with a mTOR inhibitior [36]. In our study, we report that the ULK1 inhibitor SBI, downregulates gastrin induced autophagy as well as reduces the associated migration and survival. We suggest that increased autophagic flux may be one of the several mechanisms by which gastrin induces migration, cell survival and chemoresistance. Pharmacological interference of ULK1 and PRKAA2 as therapeutic targets might provide a novel therapeutic strategy to target gastric cancer cells, which are resistant to cisplatin/5-FU treatment. In fact, the combination of HCQ with several chemotherapeutic agents is currently being investigated in clinical trials [54, 55]. These results lay the foundation for further investigation of the role of gastrin induced autophagy in survival and metastasis.

## Conclusion

Our data demonstrates that gastrin regulates autophagy via the STK11-PRKAA2-ULK1 pathway in vitro. We show that the gastrin induced migration and survival of gastric adenocarcinoma cells is partially dependent on induced autophagy. These results may contribute to a better understanding of the role of gastrin in gastric cancer. Blocking of autophagy may be a therapeutic approach for sensitizing gastric cancer cells to chemotherapy.

## Additional file

Additional file 1: Figure S1a. Expression of CCKBR in gastric adenocarcinoma cells. AGS cells have low abundance of the CCKBR, MKN45 express the CCKBR endogenously and AGS-Gr cells are stably transfected with the CCKBR. **S1b.** Negative control images of the MKN45 cells stained for the CCKBR (phase contrast, Alexa 488, Draq5). **Figure S2:** Gastrin induces autophagy. AGS-Gr (**a & b**) cells treated with gastrin (10 nM), BafA1 (100 nM) and gastrin + BafA1 for 2 and 4 h. Protein expression of MAP1LC3B-II and SQSTM1 was analyzed by immunoblotting. The images shown represent one of three independent experiments. Graphs show mean +/- SEM (P- values: *** ≤ 0.01 **≤ 0.02, and * ≤ 0.05). **Figure S3:** Negative controls (primary antibodies omitted) for MAP1LC3B (Alexa 488) and SQSTM1 (Alexa 647). **Figure S4:** Gastrin mediated survival is dependent on autophagy. (**a**): A representative cytometric plots showing AGS-Gr cells treated with BafA1 and gastrin for 18 h. Cell viability was assessed using annexin V-PI staining and flow cytometric analyses. Blocking autophagy reduces gastrin mediated survival in AGS-Gr cells. (**b**): Cell viability assessed in AGS-Gr cells treated with gastrin (10 nM) for 6- 72 h. (**c & d**): Cells treated with gastrin (2 h) and subsequently treated with increasing concentrations of cisplatin. Viability was assessed at 24 and 72 h. Results show mean +/-SD (n=3, P-values: * ≤ 0.05 ** ≤ 0.01 *** ≤ 0.001). (**e**): AGS-Gr cells treated with HCQ for 8 h. Protein expression of MAP1LC3B-II and SQSTM1 was detected by immunoblotting. (**f**) Gastrin induced autophagy is dependent on ULK1: AGS-Gr cells treated with gastrin, BafA1 and ULK1 inhibitor SBI-0206965 (10 μM) for 4 h. Protein expression of MAP1LC3B-II and SQSTM1 was detected by immunoblotting. The immunoblots represent one of three independent experiments. **Figure S5**: Inhibition of gastrin induced autophagy by Comp C. AGS-Gr cells pretreated with Compound C (10 μM) for 12 h before adding BafA1 and gastrin (4 h). Protein expression of SQSTM1 is shown by immunoblotting. The blot represents one of two independent experiments. (DOCX 2504 kb)

## Abbreviations

5-FU: 5-Flurouracil
BafA1: Bafilomycin A1
CCKBR: Cholecystokinin B receptor
CQ: Chloroquine
HCQ: Hydroxychloroquine
MAP1LC3B: Mirotubule-associated protein light chain 3 beta
mTOR: Serine/threonine-protein kinase mTOR
PRKAA2: 5'-AMP-activated protein kinase catalytic subunit alpha-1
SQSTM1: Sequestome1
STK11: Serine/threonine kinase 11
ULK1: unc-51 like autophagy activating kinase 1
