# Supplementary material for: Gastrin activates autophagy and increases migration and survival of gastric adenocarcinoma cells
Source: BMC Cancer. 2017 Jan 21;17:68. doi: 10.1186/s12885-017-3055-5 (PMC5251222; doi:10.1186/s12885-017-3055-5)
Supplement: Additional file 1: Figure S1a. — Expression of CCKBR in gastric adenocarcinoma cells. AGS cells have low abundance of the CCKBR, MKN45 express the CCKBR endogenously and AGS-Gr cells are stably transfected with the CCKBR. S1b. Negative control images of the MKN45 cells stained for the CCKBR (phase contrast, Alexa 488, Draq5). Figure S2: Gastrin induces autophagy. AGS-Gr (a & b) cells treated with gastrin (10 nM), BafA1 (100 nM) and gastrin + BafA1 for 2 and 4 h. Protein expression of MAP1LC3B-II and SQSTM1 was analyzed by immunoblotting. The images shown represent one of three independent experiments. Graphs show mean +/- SEM (P- values: *** ≤ 0.01 **≤ 0.02, and * ≤ 0.05). Figure S3: Negative controls (primary antibodies omitted) for MAP1LC3B (Alexa 488) and SQSTM1 (Alexa 647). Figure S4: Gastrin mediated survival is dependent on autophagy. (a): A representative cytometric plots showing AGS-Gr cells treated with BafA1 and gastrin for 18 h. Cell viability was assessed using annexin V-PI staining and flow cytometric analyses. Blocking autophagy reduces gastrin mediated survival in AGS-Gr cells. (b): Cell viability assessed in AGS-Gr cells treated with gastrin (10 nM) for 6- 72 h. (c & d): Cells treated with gastrin (2 h) and subsequently treated with increasing concentrations of cisplatin. Viability was assessed at 24 and 72 h. Results show mean +/-SD (n=3, P-values: * ≤ 0.05 ** ≤ 0.01 *** ≤ 0.001). (e): AGS-Gr cells treated with HCQ for 8 h. Protein expression of MAP1LC3B-II and SQSTM1 was detected by immunoblotting. (f) Gastrin induced autophagy is dependent on ULK1: AGS-Gr cells treated with gastrin, BafA1 and ULK1 inhibitor SBI-0206965 (10 μM) for 4 h. Protein expression of MAP1LC3B-II and SQSTM1 was detected by immunoblotting. The immunoblots represent one of three independent experiments. Figure S5: Inhibition of gastrin induced autophagy by Comp C. AGS-Gr cells pretreated with Compound C (10 μM) for 12 h before adding BafA1 and gastrin (4 h). Protein expression of SQSTM1 is shown by immunoblot [file 12885_2017_3055_MOESM1_ESM.docx]

**Supplementary files**

**Gastrin activates autophagy and increases migration and survival of gastric adenocarcinoma cells**

**Shalini V. Rao^1,2^, Guri Solum^1^, Barbara Niederdorfer^1^, Kristin G. Nørsett^1,4^, Geir Bjørkøy^2,3^, Liv Thommesen^1,2^**

^1^Department of Cancer Research and Molecular Medicine, Norwegian University of Science and Technology (NTNU), Trondheim, Norway, ^2^Department of Technology, NTNU, Trondheim, Norway, ^3^CEMIR (Centre of Molecular Inflammation Research), NTNU, Trondheim, Norway, ^4^The Central Norway Regional Health Authority, Trondheim, Norway.

**Shalini Rao:** [**shalini.rao@ntnu.no**](mailto:shalini.rao@ntnu.no) **(Corresponding author)**

Prinsesse Kristinas gt. 1 Gastrosenteret 3. etg nord N-7006 Trondheim, Norway

**Supplementary files**

**S1a**

**
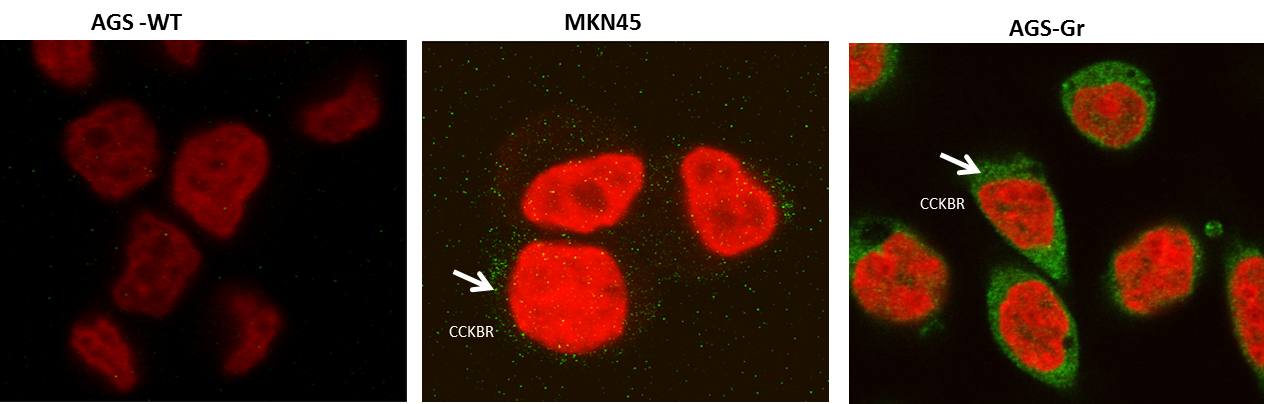
**

**S1b**

**
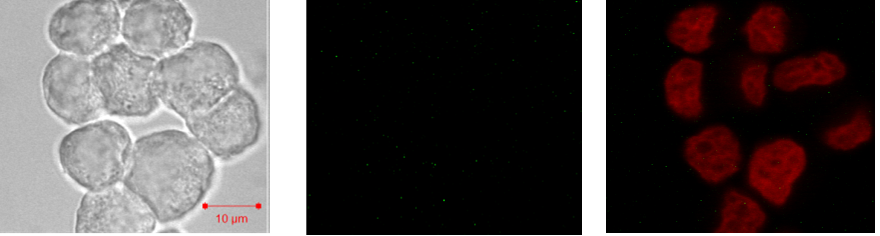
**

**
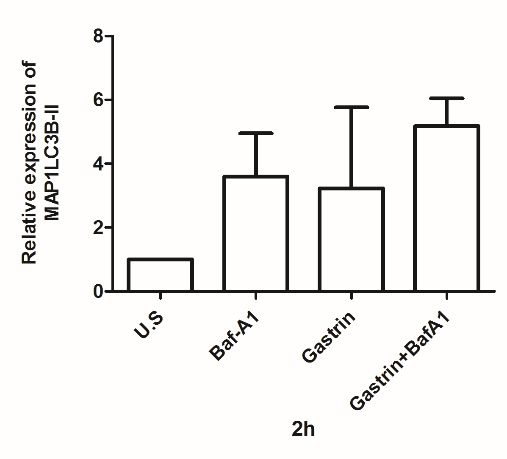

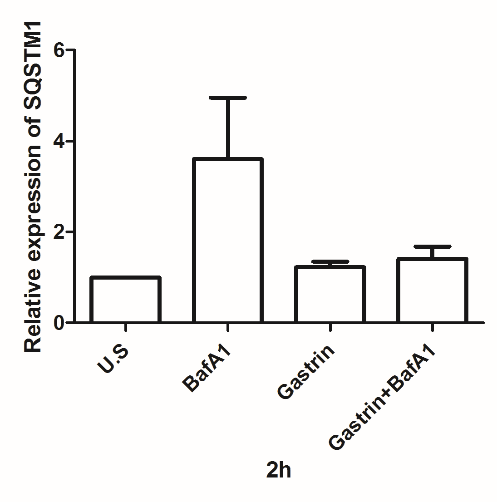
S2**

Gastrin **- - + + - - + +**

Baf A1 **- + - + - + - +**

SQSTM1

MAP1LC3BII

ACTA1

2 h 4 h

**a**


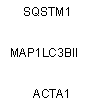

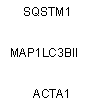


62kDa

14kDa

42kDa

62kDa

14KDa

42KDa

4h

2h

**
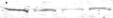
**

**
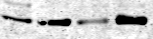
**

**
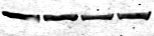
**

**b**

**
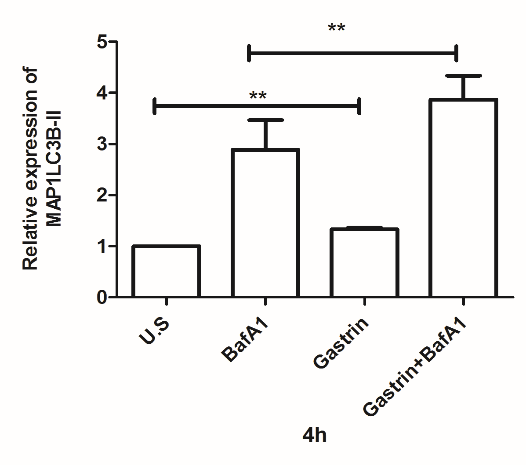

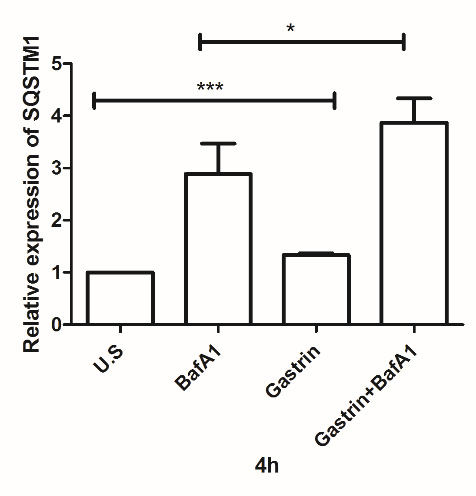

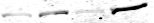
**


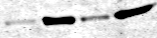


**
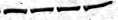
**

**Gastrin - - + +**

**Baf A1 - + - +**

**S3**


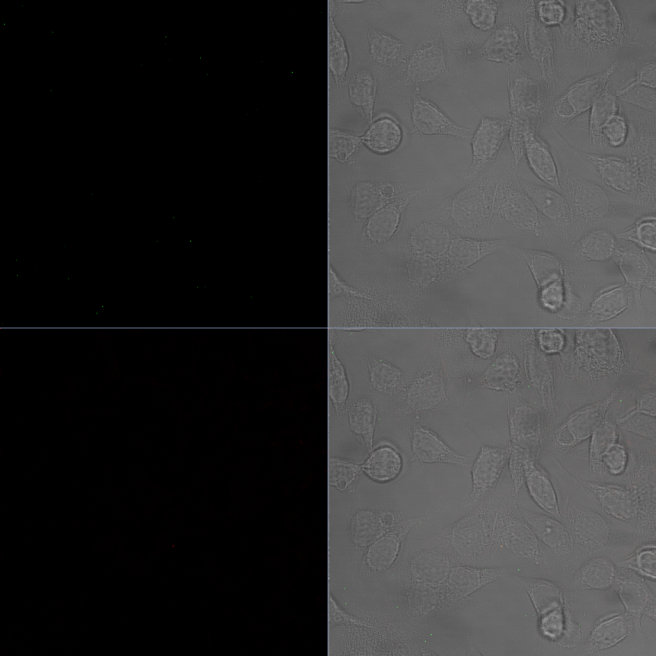

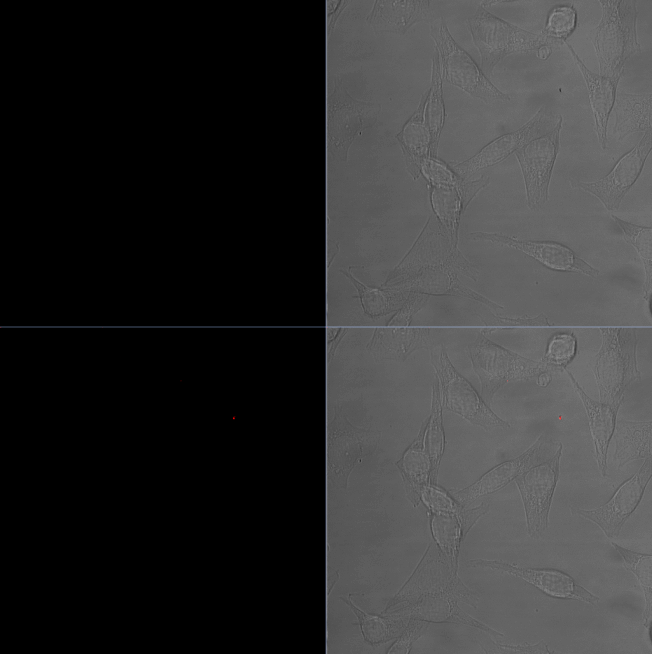
**Negative Control SQSTM1 Negative Control MAP1LC3B**

**
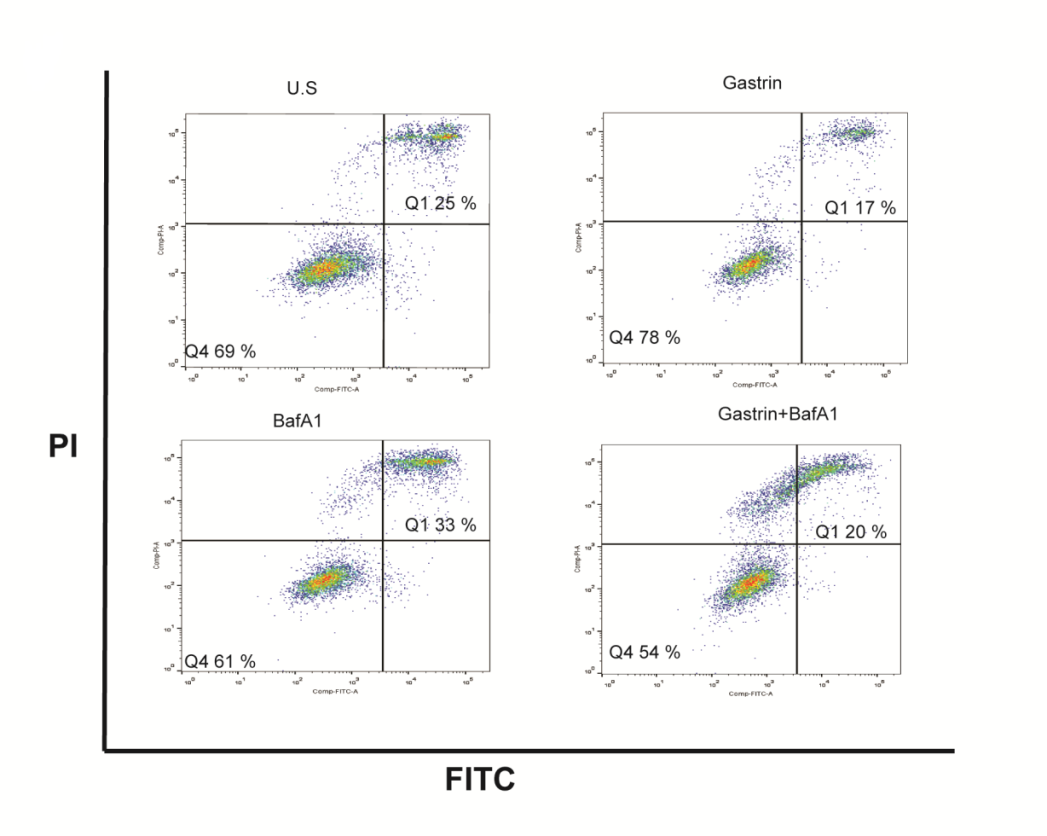
S4**

**a**

**
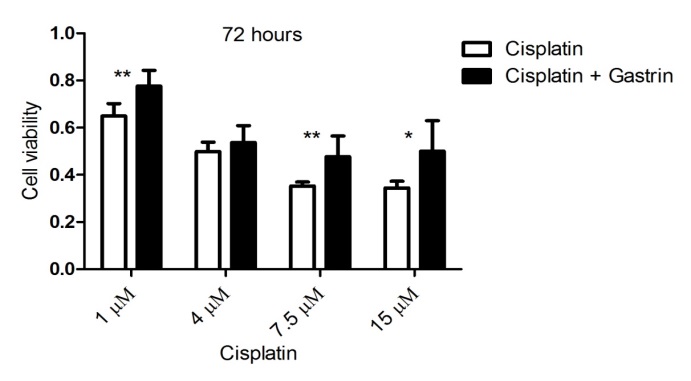
**

**d**

**c**

**b**


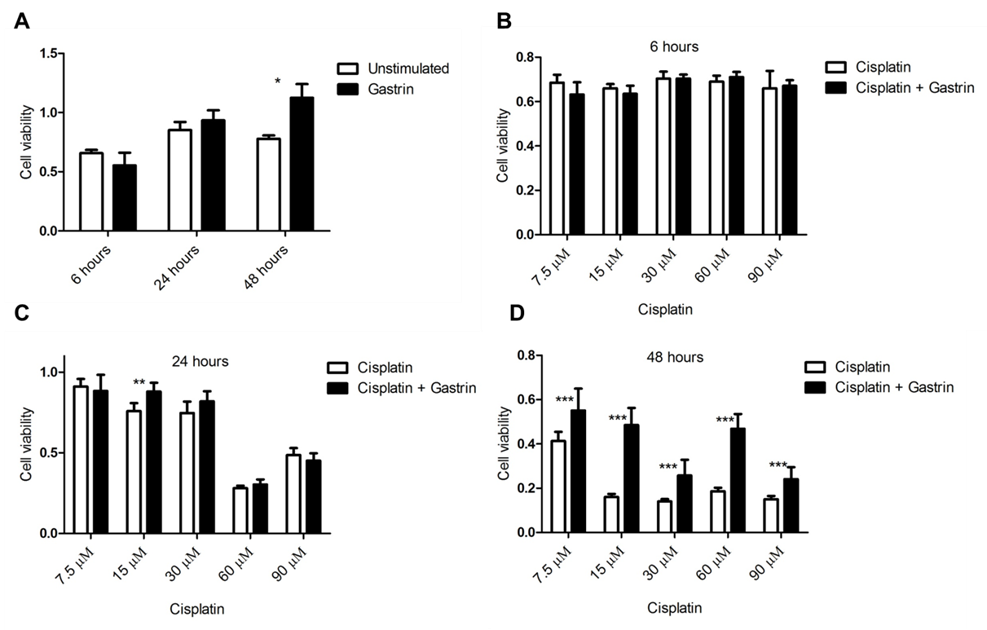

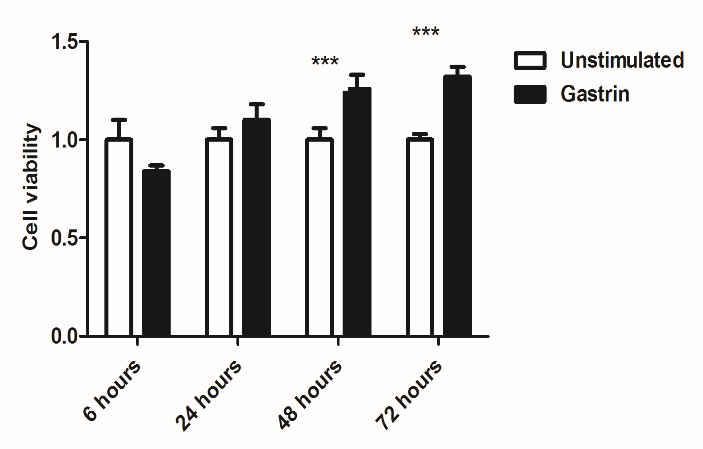


**
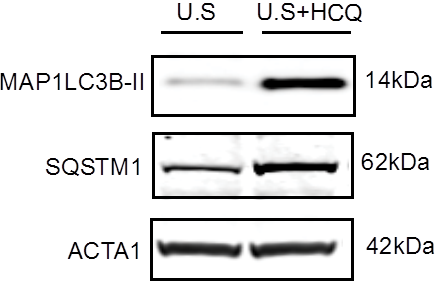
e**

**
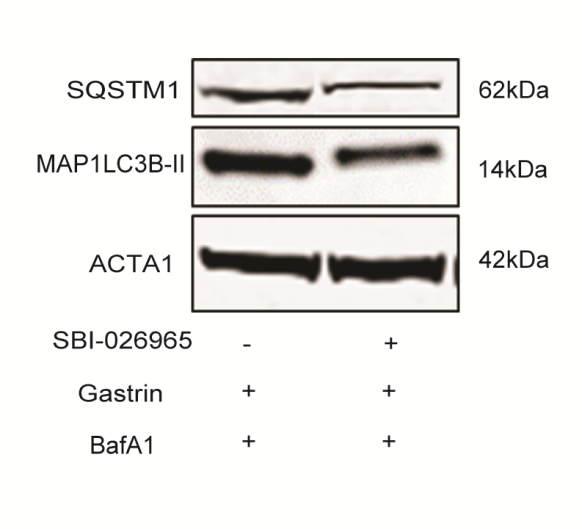
f**

**
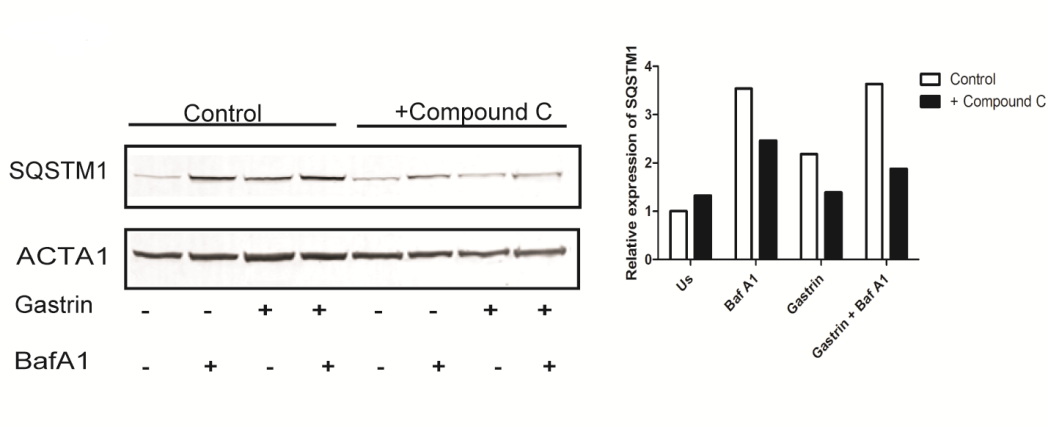
S5**
